# Computed Tomography Based Radiomics as a Predictor of Survival in Ovarian Cancer Patients: A Systematic Review

**DOI:** 10.3390/cancers13030573

**Published:** 2021-02-02

**Authors:** Stefania Rizzo, Lucia Manganaro, Miriam Dolciami, Maria Luisa Gasparri, Andrea Papadia, Filippo Del Grande

**Affiliations:** 1 Istituto di Imaging della Svizzera Italiana (IIMSI), Ente Ospedaliero Cantonale (EOC), Via Tesserete 46, 6900 Lugano, Switzerland; filippo.delgrande@eoc.ch; 2 Facoltà di Scienze Biomediche, Università della Svizzera Italiana, Via Buffi 13, 6900 Lugano, Switzerland; marialuisa.gasparri@eoc.ch (M.L.G.); andrea.papadia@eoc.ch (A.P.); 3 Department of Radiological, Oncological and Pathological Sciences, Sapienza University of Rome, 00185 Rome, Italy; lucia.manganaro@uniroma1.it (L.M.); miriam.dolciami@uniroma1.it (M.D.); 4 Department of Gynecology and Obstetrics, Ente Ospedaliero Cantonale (EOC), Via Tesserete 46, 6900 Lugano, Switzerland

**Keywords:** ovarian cancer, radiomics, overall survival, progression free survival, heterogeneity

## Abstract

**Simple Summary:**

Ovarian cancer represents the most lethal gynecological malignancy. Since many new drugs have been recently introduced as adjunctive treatments for this pathology, an early prediction of outcome might be helpful to further improve outcomes. Radiomics represents a recent advancement, relying on extraction of quantitative features from imaging examinations. Indeed, clinical images, such as computed tomography images, may contain quantitative information, reflecting the underlying pathophysiology of a tumoral tissue. Radiomic analyses can be performed in tumor regions and metastatic lesions, as well as in normal tissues. The radiomic process relies on quantitative features, usually extracted by dedicated software, and then culminates in analysis and model building, according to a defined clinical question. This systematic review aims to evaluate association between radiomics based on computed tomography images and survival (in terms of overall survival and progression free survival) in ovarian cancer patients.

**Abstract:**

The objective of this systematic review was to assess the results of radiomics for prediction of overall survival (OS) and progression free survival (PFS) in ovarian cancer (OC) patients. A secondary objective was to evaluate the findings of papers that based their analyses on inter-site heterogeneity. This systematic review was conducted according to the PRISMA statement. After the initial retrieval of 145 articles, the final systematic review comprised six articles. Association between radiomic features and OS was evaluated in 3/6 studies (50%); all articles showed a significant association between radiomic features and OS. Association with PFS was evaluated in 5/6 (83%) articles; the period of follow-up ranged between six and 36 months. All the articles showed significant association between radiomic models and PFS. Inter-site textural features were used for analysis in 2/6 (33%) articles. They demonstrated that high levels of inter-site textural heterogeneity were significantly associated with incomplete surgical resection in breast cancer gene-negative patients, and that lower heterogeneity was associated with complete resectability. There were some differences among papers in methodology; for example, only 3/6 (50%) articles included validation cohorts. In conclusion, radiomic models have demonstrated promising results as predictors of survival in OC patients, although larger studies are needed to allow clinical applicability.

## 1. Introduction 

Ovarian cancer (OC) represents one of the most lethal gynecological cancers in the world, accounting for about 14,000 deaths in 2020 in the US [1]. Computed Tomography (CT) is part of the standard pre-treatment evaluation of OC patients to evaluate the spread of the disease [2]. Traditionally, radiologists subjectively evaluate clinical images, based on their training and experience, to provide a diagnosis or an assessment of a clinical state [3].

In the last few years, personalized and precision medicine have begun paving the way towards tailored and individualized treatments, based on the increasing knowledge of the tumoral microenvironment, at a microbiological and molecular level. Radiomics represents a recently introduced translational field of research, aiming to find associations between quantitative information extracted from imaging examinations and clinical data to support evidence-based clinical decision-making [4].

The concept underlying radiomics is that clinical images, such as CT images, may contain quantitative information, reflecting the underlying pathophysiology of a tumoral tissue [5]. Radiomic analyses can be performed in tumor regions and metastatic lesions, as well as in normal tissues [4,6]. The radiomic process relies on quantitative features, usually extracted by dedicated software, and then culminates in analysis and model building, according to a defined clinical question. Despite some promising results of radiomics as a predictor of prognosis in cancer patients [5,7,8,9], there are many concerns about the applicability of these models and ethical issues [10,11]. Indeed, the main limitations of most radiomic studies are different approaches to the extraction of features, in terms of software, as well as in terms of tissues considered for feature extraction (the tumor itself or the metastases), the high number of features despite the small number of patients included, and the lack of validation cohorts [9,12].

So far, a few narrative reviews about the role of radiomics in evaluation of ovarian cancer patients have been published [3,13,14,15,16]. To the best of our knowledge, no systematic review has specifically addressed the role of radiomics in evaluation of survival in OC patients.

The main objective of this systematic review was to assess the results of radiomics for prediction of overall survival (OS) and progression free survival (PFS) in OC patients. A secondary objective was to evaluate the findings of papers that based their analyses on inter-site heterogeneity.

## 2. Methods

This systematic review was conducted according to the Preferred Reporting Items for Systematic Reviews and Meta-analysis for Diagnostic Test Accuracy (PRISMA-DTA) statement [17], which describes an evidence-based minimum set of items for reporting in systematic reviews and meta-analyses of diagnostic studies.

### 2.1. Search Strategy

Two authors (SR and LM) performed a comprehensive computer literature search of the electronic databases PubMed, Cochrane, and Web of Science to find primary publications evaluating CT based texture analysis in ovarian cancer. No beginning date limit or language restrictions were used; the literature search was last updated on 31st October 2020; the search was expanded by also screening the references of the retrieved articles for additional potentially eligible studies.

### 2.2. Study Selection

The search terms consisted of ((ovary) OR (ovarian)) AND ((radiomic) OR (radiomics) OR (texture) OR (textural)) AND ((survival) OR (prognosis) OR (prognostic) OR (predictive) OR (predicting) OR (prediction)). Articles in which CT-based texture analysis and radiomics were employed for prognostic purposes of ovarian cancer were obtained in full for further evaluation. Studies were excluded if they were case reports, conference abstracts, reviews, or short communications because they do not provide sufficient information to assess the methodological quality. Uncertainties were resolved in consensus.

### 2.3. Data Extraction

For each eligible article, information was collected concerning the basic study (authors, year of publication, country of origin, journal impact factor, quartile ranking of the journal, study design), patient characteristics (number of patients evaluated, mean/median age, stage according to the International Federation of Gynecology and Obstetrics (FIGO) staging, OS and PFS, including median follow-up in months), technical aspects (presence of a validation group, extraction of features exclusively from the ovaries or from more than one site of disease, number of features included in the final model, categories and names of features included, software used for segmentation and for feature extraction, CT scan manufacturer and protocol of acquisition, ROI tracing).

### 2.4. Quality Assessment

The overall quality of the included studies was critically evaluated based on the revised “Quality Assessment of Diagnostic Accuracy Studies” tool (QUADAS-2) [18]. This tool comprises four domains (patient selection, index test, reference standard, and flow and timing) and each domain was assessed in terms of risk of bias, and a graph was constructed appropriately.

## 3. Results

### 3.1. Literature Search

**Figure 1:**
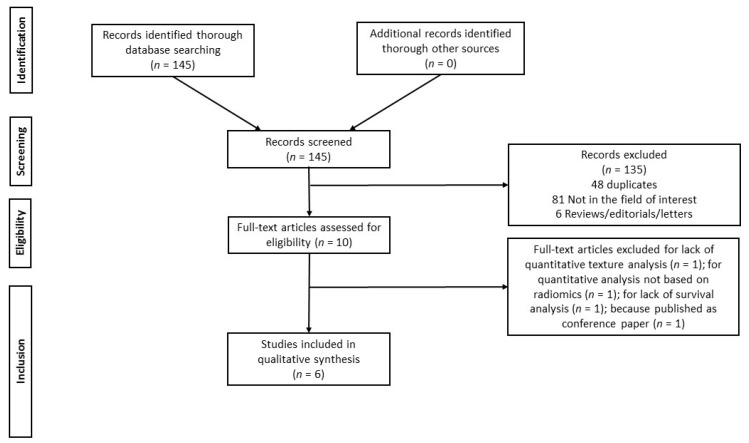
Study Selection Flowchart.

The initial search yielded 145 articles, all in English. According to inclusion and exclusion criteria, six full-text articles were included in this systematic review. Details about the literature search results are reported in Figure 1.

Given the small number of papers included, and the heterogeneity of the quantitative analyses performed as well as of the results, a metanalysis for pooled data was not performed.

### 3.2. Basic Study and Patient Characteristics

Six articles evaluating the association between CT based radiomics and survival in OC patients were selected [18,19,20,21,22,23,24]. The selected articles were published between 2017 and 2019 by researchers from Europe (*n* = 2), US (*n* = 3), and China (*n* = 1). All the studies were retrospective; the number of patients included ranged between 38 and 364; the mean/median age of patients ranged between 50 and 75 years; most of the patients included had a FIGO stage III or IV, median follow-up ranged between 23.1 and 59 months (Table 1).

### 3.3. Methodological and Technical Aspects of the Included Studies

Three out of six (50%) studies included one or more validation cohorts [19,23,24], 2/3 (33%) included both internal and external validation [19,23]. The number of features included in the prognostic models, categories and names of the features, software used for segmentation, and feature extraction are summarized in Table 2. Technical details about CT acquisition and ROI tracing of the included studies are summarized in Table 3.

**Figure 2:**
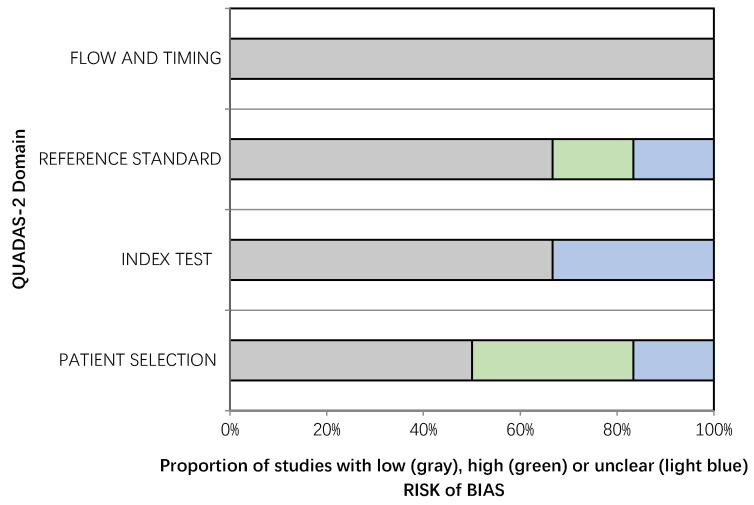
Overall Quality Assessment of the Studies Included in the Systematic Review, According to the QUADAS-2 Tool.

The overall quality assessment of the studies is reported in Figure 2.

### 3.4. Main Findings

#### 3.4.1. Overall Survival

Association between radiomic features and OS was evaluated in 3/6 (50%) studies; the median period of follow-up, where declared, ranged between 53 and 59 months. All the articles showed a significant association between radiomic features and OS. Specifically, Lu et al. drew up a prognostic score of four features, named radiomic prognostic vector (RPV), selected from a larger group of 42 features by using least absolute shrinkage and selection operator (LASSO). Using the RPV, OS differences were confirmed in two independent validation datasets; furthermore, the addition of RPV improved the clinically available prognostic methods (stage, age, and postoperative residual disease) in all three datasets, as measured by the concordance index [19].

The other two studies based their model on features indicating inter-site similarity. Meier et al. demonstrated that smaller values of similarity entropy (SE) were significantly associated with longer OS (*p* = 0.014) [20]; Vargas et al. demonstrated that SE, similarity level cluster shade (SCS), and inter-site similarity level cluster prominence (SCP) were associated with shorter OS (*p* = 0.02, 0.017 and 0.028, respectively) [22].

#### 3.4.2. Progression Free Survival

Association with PFS was evaluated in 5/6 (83%) studies; the median period of follow-up, where declared, ranged between 26 and 59 months. Lu et al. [19] demonstrated that the above-mentioned RPV was significantly associated also with PFS. Meier et al. [20] demonstrated that inter-site textural heterogeneity metrics, such as lower values of inter-site cluster variance (SCV) and inter-site cluster prominence (SCP), were significantly associated with longer PFS, with *p*-values of 0.006 and 0.021, respectively.

Rizzo et al. [21] showed that at the univariate analysis three features—fitting to the gray level run length matrix (GLRLM), to the shape 3D features and to the gray level co-occurrence matrix (GLCM)—were significantly associated with disease progression at 12 months. Multivariate analysis, on the other hand, confirmed the higher risk only for the feature belonging to the shape 3D cluster. Furthermore, the authors demonstrated that a clinical-radiomic model outperformed a clinical model (*p* = 0.04), with corresponding AUC (95% CI) of 0.87 (0.76–0.97) and 0.73 (0.54–0.93), respectively.

Wei et al. [23] built a radiomic signature for association with PFS with four radiomic features. These included features belonging to the zone-size variance in the gray-level size zone matrix (GLSZM), features extracted from the wavelet transform and a first-order statistics (FOS) feature. The discrimination accuracy of the radiomic nomogram for predicting 3-year recurrence risk was 88.9% (95% CI, 85.8–92.5%), whereas it was only 73.7% (95% CI, 69.4–78.1%) via the clinical prognostic model alone.

Zargari et al. [24] used a particle swarm optimization (PSO) to narrow down the number of features included in the model (*n* = 11) and to give each one a different weight. The features belonged namely to the shape and density, discrete cosine transform (DCT), gray level difference method (GLDM), and wavelet groups. Among these, the features in the shape and density group showed greater weights than others in the DCT and GLDM groups, indicating the importance of the features coming from the frequency domain analysis in establishing the optimal synthetic feature (*p*-values between 0.002 and 0.017).

#### 3.4.3. Radiomic Similarity

Analysis was based on radiomic features extracted from several sites of disease in 3/6 (50%) papers; however, only 2/3 (66%) based their analysis on the inter-site textural heterogeneity metrics. Meier et al. showed that high levels of three inter-site textural heterogeneity metrics were significantly associated with incomplete surgical resection in breast cancer gene (BRCA)-negative patients, but not in BRCA-positive patients. Comparison of texture heterogeneity metrics and surgical resection status demonstrated significant association only for the SCV, suggesting that lower heterogeneity was associated with complete resectability. On the other hand, the evaluated texture metrics were not able to distinguish between BRCA mutation carriers and non-mutation carriers [20].

Vargas et al. confirmed that lower heterogeneity was associated with complete resectability. However, they did not find significant associations between inter-site texture heterogeneity metrics and the cancer genome atlas (TCGA) classification of the OC subtype, when grouped by mesenchymal versus non-mesenchymal subtypes, with the exception of inter-site homogeneity [22].

All the above-mentioned findings are summarized in Table 4.

## 4. Discussion

Since OC presents no specific symptoms in the early stage of the disease, over 75% of women are diagnosed at advanced stages with a 5-year survival of 15−25% [1].

The gold standard for OC is a primary cytoreduction followed by platinum-based adjuvant chemotherapy. It has been clearly demonstrated that the absence of residual tumor (RT) at the end of the surgical cytoreduction is the most important factor positively impacting on patient survival [25,26]. An increase in 10% of complete resection (RT = 0) is associated with a 5% improvement in patient OS [27]. Unfortunately, no effective tools are available for a preoperative selection of patients at diagnosis in whom complete resection will be achieved, and who will, therefore, have a better OS. In many institutions, the resectability is evaluated laparoscopically with an easy and reproducible score [28]. When a complete resection seems to be achievable, the laparoscopy is converted to a laparotomy for a primary cytoreduction. When a complete resection does not seem to be achievable, the laparotomy is omitted in favor of neoadjuvant chemotherapy followed by interval debulking surgery and adjuvant chemotherapy.

Despite the high responsiveness to platinum-based regimens, up to 80% of patients with advanced OC experience relapses with a median progression-free survival of 12–18 months [29].

OC is a complex disease, with different histological features and molecular expression. Implementation of biobanking and analysis of tumor samples is considered a promising way to identify markers predictive of response [30]. Among these new methods, radiomics may represent a noninvasive pre-operative tool for stratification of OC patients according to outcome.

Radiomic analysis is a multistep process, where each single step is important for the robustness of the final result. For example, segmentation can be performed with different software programs and can rely on manual, semi-automatic, or automatic contouring. This is both challenging, because many tumors have unclear borders, and contentious because there is no consensus on the need to seek either the ground truth or the reproducibility of image segmentation [4,5]. In this systematic review, only 2/6 papers used the same software for segmentation, thus, demonstrating a moderate variability.

Likewise, the software used for feature extraction showed an even larger variability, because none of the six papers used the same one. This partially accounts for the different results among the articles. Indeed, the radiomic features belong to different categories and not all the software programs extract all the categories. Principally, the features may belong to the following orders of categories. The so-called first order statistics features describe the distribution of individual voxel values without concern for spatial relationships, such as mean, median, maximum, minimum, entropy, skewness, and kurtosis of the histogram of values. The first-order statistics features also include the shape features, which describe the shape of the traced ROI and its geometric properties.

The second order statistics features describe textural features, meaning the statistical inter-relationships between voxels [4], and in radiomics this can readily provide a measure of intratumoral heterogeneity. Examples of these features are all the matrices based on different gray levels, such as the GLCM, GLRLM, and so on. Higher order statistics features are generated by statistical methods after imposing filter grids on the image to extract repetitive or nonrepetitive patterns. These include fractal analyses, Minkowski functionals, and wavelets [4]. Once the features have been extracted, a reduction in number is needed, to avoid redundancy. Furthermore, the stability and reproducibility of the model must be assessed and then confirmed before applying a predictive model in a clinical setting. Indeed, model fitting is usually optimal in the training set for model building, whereas its validation in external cohorts may provide more reliable fitting estimates [31]. The first validation is usually internal cross-validation, but the reference standard should be validation in independent cohorts, ideally prospectively collected within clinical trials [9].

The CT based radiomic association with OS in OC patients was partially addressed by 3/6 articles included in this review. Although all of them demonstrated a significant association of the radiomic model with OS, the features were extracted from the ovarian masses in one article that also included validation cohorts [19], whereas they were extracted from the peritoneal carcinomatosis in 2/3 articles that based their model on the similarity indices [20,22]. These differences account for promising results, but also indicate the need for larger cohorts, possibly supplemented by external validation cohorts.

The CT based radiomic association with PFS in OC was addressed by 5/6 articles included in this review. Three out of five based their feature extraction solely on the ovarian masses [19,21,23], and 2/5 on peritoneal carcinomatosis [20,24]; 3/6 articles included validation cohorts [19,23,24]. The five articles demonstrated associations between the radiomic model and PFS; all the models included features that fell within the second order statistical features, but in 3/5 articles features from the first order statistics and from higher order statistics features were also included [21,23,24]. Furthermore, one paper based its conclusions on evaluation of inter-site heterogeneity features [20]. Noticeably, there is a wide variation in methods also for association with PFS and, consequently, the models are still not transferable as such into clinical practice.

OC is a heterogeneous disease composed of many different histologic subtypes, the most frequent being the high-grade serous, low-grade serous, endometrioid, clear cell, and mucinous type. Each histological type is associated with unique clinical etiologies, sensitivity to therapies, and molecular signatures, including diverse transcriptional regulatory programs [32,33]. Since histological sampling from different sites of disease is not feasible over a period of time, CT based radiomics may represent an optimal tool to capture the heterogeneity of the disease. For this reason, a secondary objective of this systematic review was a dedicated evaluation of papers that based their models on inter-site heterogeneity. In this review, 3/6 articles based their considerations on the extraction of features from many sites of disease [20,22,24], and among these 2/3 calculated specific similarity indices [20,24]. Based on the TCGA data, a prognostic algorithm for high grade serous ovarian cancer, known as classification of ovarian cancer (CLOVAR) with four subtypes has been defined: differentiated, immunoreactive, mesenchymal, and proliferative [32,34], where the mesenchymal subtype has demonstrated shorter survival. Vargas et al. developed 12 quantitative metrics to capture spatial inter-site imaging heterogeneity in high-grade serous ovarian cancer. The authors demonstrated that metrics capturing the differences in texture similarities across sites were associated with shorter OS [22]. Later, Meier et al. assessed the associations between inter-site texture heterogeneity parameters, survival, and BRCA status, demonstrating that high inter-site cluster variance was associated with lower PFS and OS. Furthermore, the authors demonstrated that high values of all the three metrics included were significantly associated with lower rate of complete surgical resection in BRCA-negative patients [20]. However, neither of these studies included validation of their models and the number of patients was limited (*n* = 38 in [22] and *n* = 88 in [20]); therefore, for this objective too, larger studies including validation cohorts are needed.

This review has some inherent limitations. First, the small number of papers included. This is strictly related on one hand to the novelty of the topic, and on the other to the selection of radiomics papers applied to OC, that based their analysis on CT images and had survival as their endpoint. However, these selection criteria were chosen to answer an unmet clinical need for clarification regarding the role of radiomics as a predictor of survival in OC. Second, all the studies included were retrospective, with considerable variability in number of patients included and methodology. These methodological flaws were taken into account and prevented us from going further and carrying out a metanalysis. Third, in the last few years new diversified drugs have been introduced as treatments in OC, such as Poly ADP-ribose polymerase (PARP) inhibitors, angiogenesis inhibitors and immunomodulators, that have significantly increased survival, although associated with an increase of treatment-related toxicities [35,36]. In this regard, none of the articles included evaluated whether the differences in survival were related to the introduction of new drugs. A further limitation is that, given the availability of different software, some for free download, others under payment, none of the six papers included used the same software for textures extraction. Furthermore, the radiomics extraction methodology, including mentions to normalization, creation of isotropic images and interpolation was not described in all the studies and, therefore, is not reported in this review.

## 5. Conclusions

In conclusion, radiomic models have demonstrated promising results as predictors of survival in OC patients. However, larger studies that include validation cohorts, and take into account the introduction of new drugs that may prolong survival, are needed to transfer the prediction models to clinical practice.

## Figures and Tables

**Table 1:** Basic Study and Patient Characteristics.

Authors	Year	Country	Journal Impact Factor *	Quartile *	Study Design	*N* Patients	Mean/Median Age	FIGO Stage I–II	FIGO Stage III–V	OS Evaluation (Median, Follow-Up Months)	PFS Evaluation (Median, Follow-Up Months)
Lu, H., et al. [19]	2019	UK	12.121	Q1	R	364	62	53	223	Yes (53.2)	Yes (23.1)
Meier, A., et al. [20]	2019	US	2.429	Q2	R	88	75	ND	ND	Yes (59)	Yes (59)
Rizzo, S., et al. [21]	2018	Italy	4.101	Q1	R	101	53	11	90	No	Yes (26)
Vargas, H.A., et al. [22]	2017	US	4.101	Q1	R	38	ND	0	38	Yes (56.4)	No
Wei, W., et al. [23]	2019	China	4.848	Q2	R	142	50	0	142	No	Yes (27.7)
Zargari, A., et al. [24]	2019	US	2.883	Q2	R	120	67	ND	ND	No	Yes (ND)

* According to Journal of Citation Reports 2020 (Impact factor and quartile ranking of 2019); FIGO = International Federation of Gynecology and Obstetrics; OS = Overall Survival; PFS = Progression Free Survival; R = retrospective; ND = Not declared.

**Table 2:** Methodological and Technical Aspects of the Included Studies.

Authors	Validation Group/Groups	Extraction of Features Exclusively from Ovaries	Extraction of Features from More than One Site of Disease	Number of Features Included in the Final Model	Categories and Names of Features Included	Software Used for Segmentation	Software Used for Feature Extraction
Lu, H., et al. [19]	Yes (internal and external)	Yes	No	4	Shape, density, texture and wavelet (GLRLM; NGTDM; FOS; FD)	ITK-SNAP	TextLAB 2.0
Meier, A., et al. [20]	No	No	Yes	3	Texture, Haralick (GLCM; SE; SCV; SCP)	ITK-SNAP	Matlab based
Rizzo, S., et al. [21]	No	Yes	No	3	Shape, density, texture (GLRLM; shape 3D; GLCM)	DICOM RT structure	IBEX
Vargas, H.A., et al. [22]	No	No	Yes	3	Texture, Haralick (GLCM; SE; SCS; SCP)	3D Slicer	ND
Wei, W., et al. [23]	Yes (internal and external)	Yes	No	4	Shape, texture, histogram, wavelet (FOS; GLSZM)	ITK-SNAP	Matlab based
Zargari, A., et al. [24]	Yes (internal)	No	Yes	11	Shape, density, texture, wavelet Shape and density; DCT; GLDM; Wavelet)	ND	ND

GLRLM = Gray Level Run Length Matrix; NGTDM = Neighborhood Gray Tone Difference Matrix; FOS = First Order Statistics; FD = Fractal Dimension; GLCM = Gray Level Co-occurrence Matrix; SE = Inter-site Entropy; SCV = Inter-site Cluster Variance; SCP = Inter-site Cluster Prominence; 3D = 3 dimensions; SCS = Inter-site Cluster Shade; GLSZM = Gray Level Size Zone Matrix; DCT = Discrete Cosine Transform; GLDM = Gray Level Difference Method; ND = not declared.

**Table 3:** Technical Details About CT Acquisition and ROI Tracing of the Included Studies.

Authors	CT Scan Manufacturer and Protocol (Slice Thickness; Acquisition Parameters; Contrast Bolus)	ROI Tracing (Single Slice/Volumetric; Manual/Semi-Automatic/Automatic; Single or Multi Reviewers)
Lu, H., et al. [19]	Several CT manufacturers and protocols	Volumetric; ND; 3 reviewers
Meier, A., et al. [20]	GE Medical Systems; tube voltage 120 kVp; tube current 240–400 mA; section thickness 5–7.5 mm; pitch < 1; kernel Bf40; iodinated contrast medium yes	Volumetric; manual; ND
Rizzo, S., et al. [21]	Several CT manufacturers; slice thickness 1–5 mm; tube current x rotation 58–419 mAS, reconstruction algorithm filtered back projection and iterative; iodinated contrast medium yes	Volumetric; manual; single reviewer
Vargas, H.A., et al. [22]	GE Medical Systems; tube voltage 120 kVp; tube current 240–400 mA; section thickness 5–7.5 mm; pitch < 1; iodinated contrast medium yes	Volumetric; manual; ND
Wei, W., et al. [23]	Philips Medical System, GE Medical Systems; tube voltage 120 kVp; tube current 100–500 mA; section thickness 2–5 mm; pitch < 1; iodinated contrast medium yes	Volumetric; manual; 2 reviewers
Zargari, A., et al. [24]	GE Medical Systems; tube voltage 120 kVp; tube current 100–600 mA; section thickness 5 mm; pitch 1.25; iodinated contrast medium yes	Volumetric, semi-automatic; single reviewer

CT = Computed Tomography; ROI = region of interest; ND = not declared.

**Table 4:** Main Findings of the Included Studies.

Authors	Significant Associations with OS	Significant Associations with PFS	Significant Associations with Radiomic Similarity
Lu, H., et al. [19]	Association between 4 features (RPV) and OS. RPV improved the clinical prognostic methods	Association between RPV and PFS	NP
Meier, A., et al. [20]	Association between SE and OS	Association between SCV and SCP with PFS	Association between SE, SCV, SCP and surgical resection status in BRCA-
Rizzo, S., et al. [21]	NP	Association between 3 features and 12-months recurrence. The clinical-radiomics model outperformed the clinical model.	NP
Vargas, H.A., et al. [22]	Association between SE, SCS and SCP and OS	NP	Association between heterogeneity and surgical resection status.
Wei, W., et al. [23]	NP	4 features associated with prediction of 3-year recurrence. Better performance of the radiomic model than the clinical prognostic model	NP
Zargari, A., et al. [24]	NP	Association between 11 features and PFS. Greater weights for the shape and density features	NP

OS = Overall Survival; PFS = Progression Free Survival; RPV = Radiomic Prognostic Vector; NP = Not Performed; SE = Inter-site Entropy; SCV = Inter-site Cluster Variance; SCP = Inter-site Cluster Prominence; GLRLM = Gray Level Run Length Matrix; GLCM = Gray Level Co-occurrence Matrix; 3D = 3 dimensions; SE = Inter-site Entropy; SCS = Inter-site Cluster Shade; SCP = Inter-site Cluster Prominence; TGCA: The Cancer Genome Atlas; PSO = Particle Swarm Optimization; DCT = Discrete Cosine Transform; GLDM = Gray Level Difference Method; BRCA- = Breast Cancer gene negative.

## Data Availability

No new data were created or analyzed in this study. Data sharing is not applicable to this article.
